# From the bench to the reactor: engineered filamentous fungi for biochemical and biomaterial production

**DOI:** 10.1186/s13068-025-02712-8

**Published:** 2025-11-18

**Authors:** Venkataramanan Subramanian, Meaghan J. Adler, Marcus Benyamin, Randi M. Pullen, Matthew D. Servinsky, Mark T. Kozlowski, Stephen R. Decker

**Affiliations:** 1 National Renewable Energy Laboratory, 15013 Denver West Parkway, Golden, CO 80401 USA; 2 Combat Capabilities Development Command, Army Research Laboratory, 2800 Powder Mill Rd, Adelphi, MD 20783 USA

## Abstract

Filamentous fungi can convert a wide variety of naturally occurring chemical compounds, including organic biomass and waste streams, into a range of products. They have long been used for industrial organic acid production and food preparation. In this review, we will discuss production of products such as organic acids, lipids, small molecules, enzymes, materials, and foods, and highlight advances in metabolic and protein engineering, including CRISPR-Cas9-mediated strain improvements. We discuss to what extent these products are already being made on a commercial scale, as well as what is still required to make certain promising concepts industrially and commercially relevant. Despite significant progress, the systematic application of synthetic biology to filamentous fungi remains in its infancy, with many opportunities for discovery and innovation as new strains and genetic tools are developed. The integration of fungal biotechnology into circular and bio-based economies promises to address critical challenges in waste management, resource sustainability, and the development of new materials for terrestrial and extraterrestrial applications, but requires further developments in genetic engineering and process design.

## Introduction

Filamentous fungi are intertwined with our past, present, and future. Fungi have long been used as food, with some varieties seen as delicacies. Fungi are also important food contaminants, and the presence of pathogenic fungi can have real consequences for food safety. For example, it has been hypothesized that the Salem Witch Trials may have been caused by the inadvertent consumption of rye ergot fungus by the population of Salem, Massachusetts in 1692. Rye ergot produces psychoactive compounds, and the denizens of Salem may have interpreted the effects of these psychoactive compounds as the result of witchcraft [1]. Filamentous fungi also make helpful small molecules that have medicinal effects, and provide key medicines such as antibiotics and anticancer agents. In the present, we are faced with the interconnected problems of waste management and resource requirements, so we may increasingly rely on fungus to convert waste into useful products. Finally, engineered fungi may have a role to play as humanity pushes further into the exploration of outer space. The weight that can be carried into space is very limited, meaning that many of the needs of space explorers will have to be met with localized manufacturing that can use locally-available materials such as regolith.

Filamentous fungi excel in two unique properties. They are known for secreting large quantities of extracellular enzymes that depolymerize complex substrates and for producing chemically diverse metabolites with industrial relevance. They are remarkable at producing molecules such as organic acids, lipids, secondary metabolites, and proteins, contributing to the portfolio of fungal derived products used in the pharmaceutical, chemical, and food industries. Saprophytic fungi have evolved to utilize a wide range of substances in addition to organic biomass, such as various chemical compounds and polymeric molecules. The diverse environments in which fungi live require many different adaptations including the production of diverse secondary metabolites, which means there is a potential treasure trove of metabolic products yet to be discovered. This ability to generate a diverse array of value-added products from cost effective substrates such as agricultural, forestry, and paper industry wastes is economically and ecologically appealing. Aside from fungi themselves being useful as chemical and protein factories, the mycelium of fungi is also potentially useful as a building material. The mycelium is the network of hyphae that roots the fungus through the soil, gives it structure, secretes enzymes and enables digesting substrates for energy.

Filamentous fungi break down organic biomass and other substances by digesting them externally and then uptake the resulting material building blocks and simpler sugars such as monosaccharides. External digestion is possible due to efficient protein secretion pathways that release enzymes such as lipases, cellulases, and pectinases into their environment [2]. Secreted enzymes, organic acids, lipids, and secondary metabolites are optimally produced at differing stages of fungal growth and within varying fungal morphologies, which has an important role in the optimization of production and secretion for industrial production [3]. It has recently been noted that various parameters in process development directly impact process productivity for filamentous fungi [4], which has sparked experimental efforts to implement design control over fungal morphology into process development, such as the use of titanate microparticles in media resulting in up to seven-fold increase in glucoamylase activity in both batch and fed-batch cultivation [5]. There is mounting evidence that fungi are naturally evolving to degrade synthetic polymers and molecules such as plastics [6], and herbicides, and pesticides [7]. In combination with modern enzyme and metabolic pathway engineering [8, 9], and advances in domesticating and genetically modifying heretofore unmodified strains [10], an opportunity is emerging to leverage fungi to bioremediate pollutants into valorized compounds, further elevating their value and utility in the bioeconomy.

**Fig. 1:**
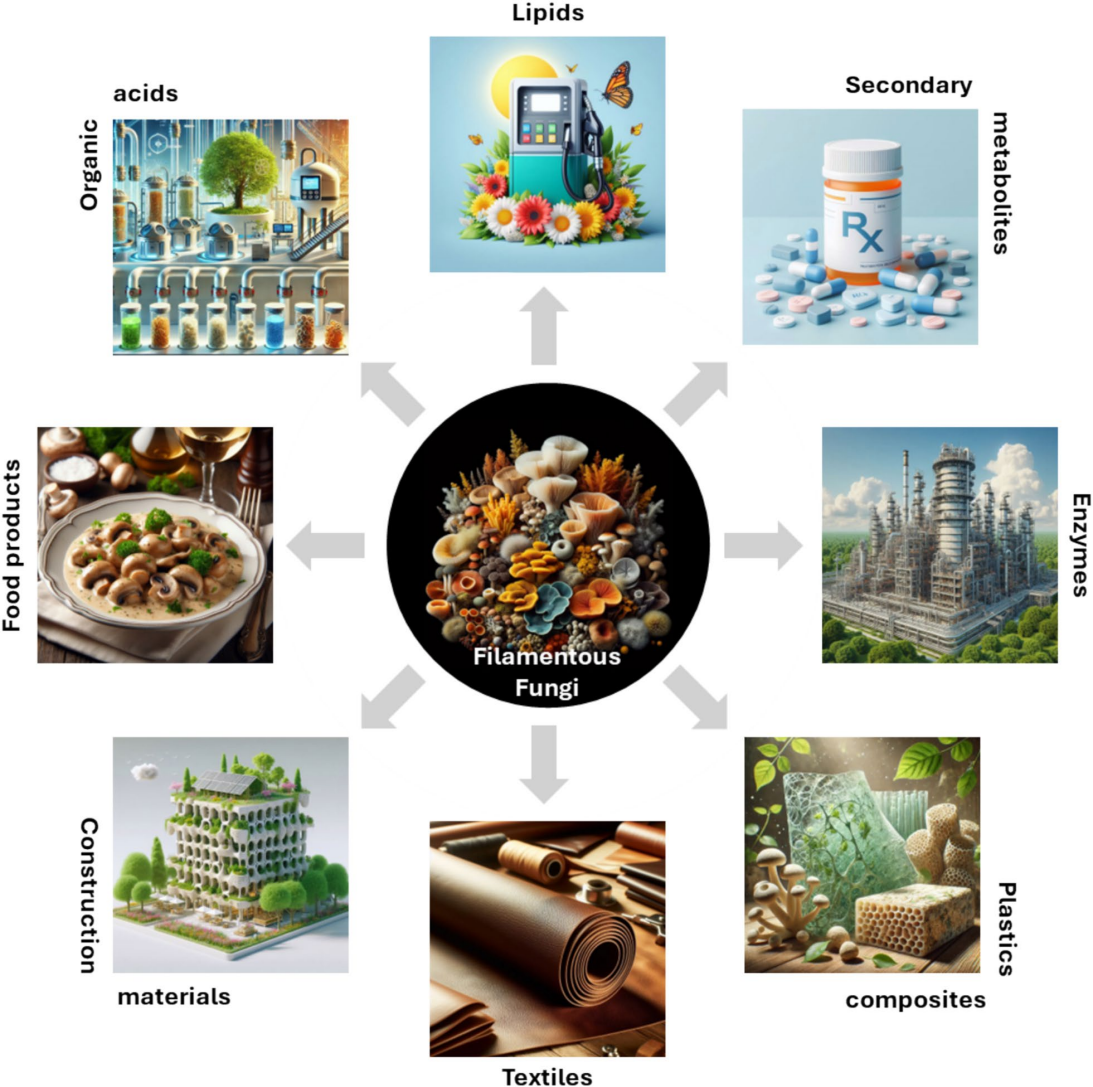
The many possible applications of filamentous fungi. Figure was generated using Microsoft Designer

Despite recent advances in metabolic and protein engineering, the genetics of filamentous fungi are still largely unexplored. In 2016, only 50–60% of genes in available filamentous fungi genomes had a predicted function, of which only 2–10% had their function confirmed by experimental study [2, 11]. This knowledge gap has spurred increasing interest in synthetic biology research to find ways to expand access to the wide range of molecules and production pathways that are unique to filamentous fungi. Those production pathways are currently used on an industrial scale to produce organic acids, lipids for biofuels, secondary metabolites for pharmaceutical development, enzymes, and proteins such as pectinases, lipases, phytases, and proteases. There is also increasing interest in using filamentous fungi for sustainable, circular-economy applications such as renewable fabrics, alternative sources of food, and alternative sources of building materials. A summary of the various applications of fungi, inspired by biotechnology is provided in Fig. 1. In this review, we will discuss the production of useful molecules and proteins by fungi, as well as using the structural properties of fungal mycelium to make new types of fabrics, materials, and food products.

## Fungi for biomolecule production

Industrial scale production of biomolecules from alternative carbon sources such as lignocellulose or organic wastes is a major objective of a biorefinery [12]. Filamentous fungi have several advantages for biorefinery processing including the ability to breakdown complex organic waste feedstocks such as lignocellulose, utilize the resultant diverse sugars, and produce a range of useful biomolecules [13, 14]. Large-scale production and extraction of organic acids, lipids, and pharmaceuticals from renewable resources such as lignocellulosic biomass will ultimately lower dependence on non-renewable, petroleum-based materials. In this section, we discuss production of organic acids, lipids, enzymes, and other secondary metabolites. Some of the examples that we share are already in industrial use, whereas others are still in a proof-of-concept or pilot phase. We also discuss the production of enzymes for degradation of plant biomass, to better enable the conversion of lignocellulosic wastes into industrial feedstock.

### Organic acid production

Filamentous fungi can produce a wide variety of organic acids which are commonly used as building blocks for food, medicine, and pharmaceuticals. Researchers speculate that filamentous fungi produce large amounts of acid to gain competitive advantage over less acid-tolerant organisms sharing the environment [15]. Pleissner et al. detailed production of economically valuable organic acids: succinic acid, lactic acid, acrylic acid, and lactobionic acid from renewable residues [16]. Many filamentous fungal species have been investigated as potential cost-effective routes to maintaining an industrial scale supply of valuable organic acids (Table 1). In one study, among 66 strains that were screened for production of organic acids, one-third of them turned out to belong to genus *Aspergillus* [15]. *Aspergillus niger* is well-known as a prolific producer of organic acids and has been studied for metabolic engineering targets to increase citric acid production [17, 18]. Tong et al. extensively reviewed recent advances in metabolic engineering and clustered regularly interspaced short palindromic repeats (CRISPR)/Cas9 system strategies to improve citric acid strain development for *A. niger* [19]. A combination of systems biology tools and genome scale metabolic modeling revealed multiple targets for strain improvements in this organism. The targets included improving carbon utilization, reducing feedback inhibition of enzymes to increase carbon flux, introducing alternate respiratory chain mechanisms, and increasing substrate uptake via engineering of cell surface transporters. Several of these critical genetic alterations require high-throughput and high efficiency genome editing tools, such as CRISPR/Cas9 to screen for strain improvement [19, 20]. Upton et al. integrated genome-scale metabolic models, dynamic modeling, and evolutionary algorithms for in silico evolution of *A. niger* ATCC1015 predicting multiple targets that could lead to maximizing productivity of organic acids while also revealing the balance between organic acid- and biomass-production, an implication highly relevant to industrial scale production of these products [21].

**Table 1:** Selected organic acids produced by wild-type or engineered fungi

Organic acid	Titer (g/L)	Organism	Conditions	Ref.	Mechanism of elevated production
Malic	165	*A. oryzae*	Glucose, pH 6 (constant)	[36, 37]	Overexpression of TCA cycle genes
Citric	140	*A. niger*	Molasses, pH 2 (final)	[38]	High nitrogen via molasses
Fumaric	107	*R. arrhizus NRRL 2582*	Glucose, pH 6 (final)	[39]	Nitrogen and neutralizing agent (CaCO_3_) limited conditions with high glucose
Itaconic	90	*A. terreus DSM 23081*	Glucose pH 1.7 (final)	[40]	Elevated phosphate conditions (0.1 g/L KH_2_PO_4_) and extended incubation period (13 days)
Lactic	65	*R. oryzae*	Corncob hydrolysate,	[38]	strain adaptation and fermentation optimization
Succinic	61	*T. reesei, A. niger, P. chrysosporium* co-culture	Soybean hull, birch wood chips	[41]	Use of a consortium of fungi and sequential fermentation stages
Gluconic	57	*A. niger CBS120.49*	Sucrose, pH 6 (constant)	[36, 42]	Various carbon sources
Oxalic	38	*A. niger CBS 120.49*	Sucrose, pH 6 (constant)	[36, 42]	pH and carbon source variation
Itaconic	30	*A. niger*	Glucose, pH 2.3 (final)	[43]	Deletion of *ictA* and *ichA* genes
Succinic	23	*A. niger*	Glucose, pH 5.5	[44]	Deletion of the *gox* and *oah* genes
L-tartaric	21.7	*P. chrysogenum*	Waste winery grapes, pH 4	[45]	Limited media optimization
Formic	3.3	*A. flavipes* *(BRFM456)*	Glucose, pH 5.5. (initial)	[15]	Industry relevant growth media
Lactic	3.14	*R. oryzae*	Potato peel waste	[46, 47]	Optimized loading rate and particle size
Lactobionic	3.1	*Pycnoporus sp. SYBC-L10*	Cellulose/soybean meal culture + lactose, pH 5.0	[48]	Lactose was added after growth and converted by produced enzymes

Production of organic acids in popular filamentous fungal species with well-annotated genomes can lead to higher titers than current industry standard strains. For example, malic acid is a four-carbon dicarboxylic acid, which is one of the top 12 building block chemicals identified by the US Department of Energy, with applications in food, textile, and pharmaceutical industries [22]. Malic acid is currently industrially-produced chemically primarily using n-butane as a starting material, leading to a racemic mixture [23], but malic acid production has been reported in fungi such as *A. flavus*, *A. oryzae*, *Ustilago trichophora* and *Myceliopthora thermophila,* with yields reported as high as 195 g/L using *U. trichophora* [24–26]. The advantages of biologically-mediated production are that only one enantiomer (L-malic acid) will be produced, and that waste substrates can be used. However, there are several important barriers that must be overcome for industrial-scale production of L-malic acid by fungi to become viable. First, several organisms that produce malic acid in high yield, fungi such as *A. flavus* also produce mycotoxins are designated as Biosafety Level 2 level organism(s), and would therefore require additional precautions to enable safe usage and production at an industrial scale. Availability and the use of molecular tools could largely mitigate these hurdles. However, for fungal malic acid production to be economically competitive, the yields obtained have to be improved. The highest malic acid production has been achieved by increasing the carbon flux of glycolytic pathway by overexpressing four genes coding for glucose transporter (MstC), hexokinase (Hk), 6-phosphofructo-2-kinase (Pfk), and pyruvate kinase (Pk) in *A. niger* [24], resulting in fed batch titer of 201.13 g/L from a 1.5 L fermention, highlighting the importance of synthetic biology in organic acid production*.*

Similarly, itaconic acid (IA) is another organic acid that has applications in superabsorbent polymers, thermoplastics, artificial glass, synthetic latex, and as an antimicrobial agent and component of antimicrobial polymers [15, 27–29]. It also falls among the top 12 most promising building blocks for a bio-based economy [22]. However, the cost of production for this bio-based alternative currently exceeds that of petroleum-based polyacrylic acid, preventing it from being a competitive substitute [2, 30]. Lowering the production cost of IA is therefore a key priority of synthetic biology. The current cost of itaconic acid is approximately $1500/metric ton (https://www.imarcgroup.com/itaconic-acid-pricing-report), whereas polyacrylic acid is approximately $1328/metric ton (https://www.imarcgroup.com/acrylic-acid-pricing-report), meaning that if the cost of itaconic acid can be reduced by about 10%, it may become a direct replacement for polyacrylic acid. Lowering the cost of biologically-produced itaconic acid could be accomplished through cheaper processing steps, increased yields from the fungi, use of cheaper feedstocks, or some combination of all three.

**Fig. 2:**
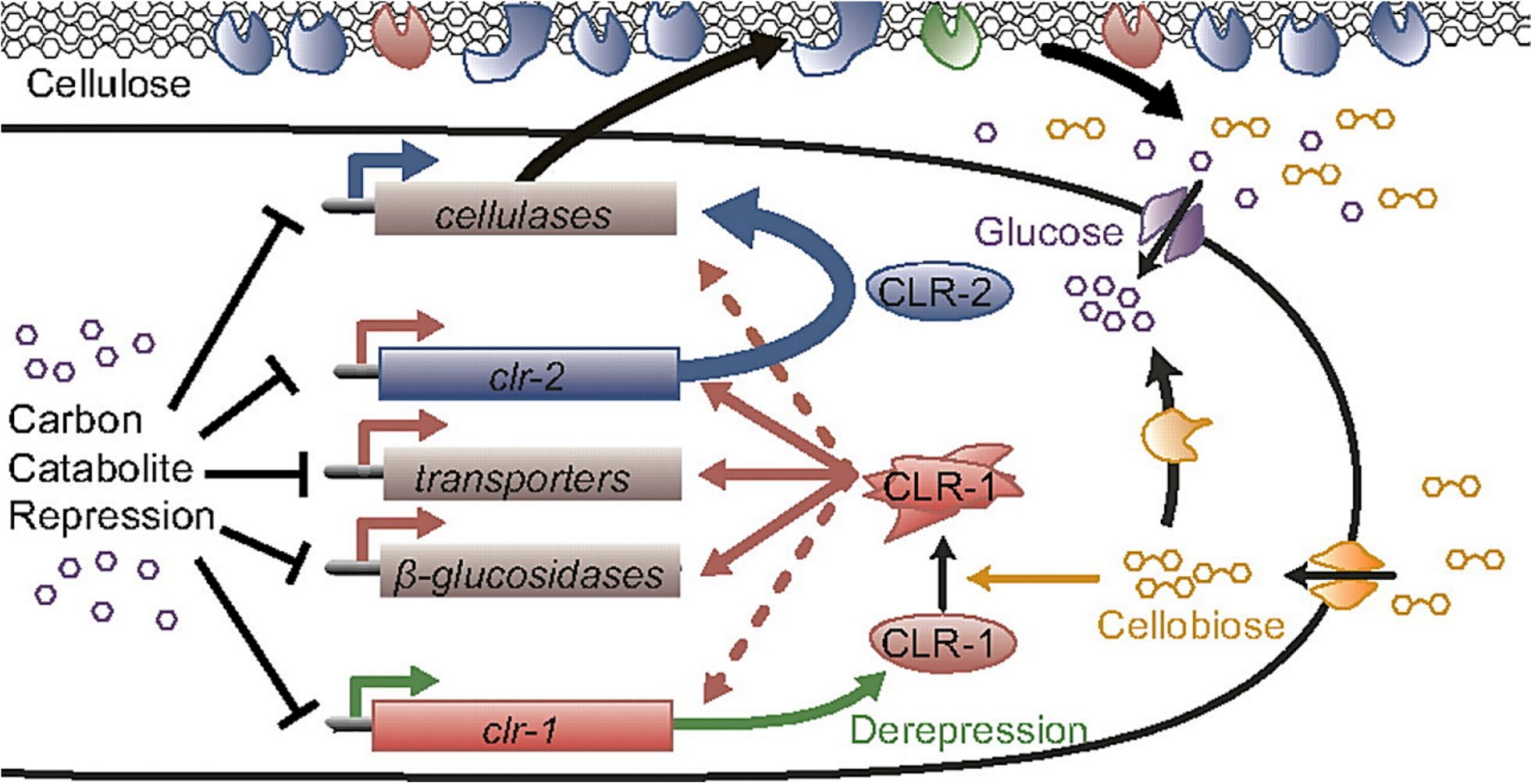
Illustration of the carbon catabolite repression (CCR) cycle, a common initial target for genetic engineering [35]. CLR; transcription factor in N. crassa that regulate breakdown of cellulose. Reproduced from the work of Coradetti and co-workers

Industrial scale production from alternative carbon sources such as lignocellulose or organic wastes can be impacted by genetic engineering of the carbon catabolite repression (CCR) system, so that filamentous fungi more easily utilize sugars from biomass wastes [31]. The CCR system mediates utilization of preferred carbon sources, like glucose, until the resource is exhausted. Once that happens, the transcription factor CreA signals the transition to a less-preferred carbon source [32, 33]. The CCR system is a common initial target for genetic engineering [34], and the combined effect of genetically engineering metabolic production pathways and the CCR pathway will most likely result in greater titers than possible with wild-type organisms. A diagram of the CCR cycle in filamentous fungi from the work of Coradetti and co-workers is reproduced in Fig. 2. [35]

Large-scale production and extraction of organic acids from renewable resources such as lignocellulosic biomass, will ultimately lower dependence on non-renewable, petroleum-based materials. While promising, more work is needed to increase the efficiency and cost of production for fungal-derived organic acids to be marketable alternatives. Another limitation is that organic acids are produced aerobically by fungi, and therefore any bioreactor methods must consider the need for a sufficient supply of oxygen, and this impacts yields. Research in the field of transition to industrial production is somewhat hampered by the fact that negative results of pilot facilities aren’t published, and successful adaptation to industry is usually not published either, as that information is kept proprietary. Academic process development centers may play a key role in moving proof-of-concept strains to industry.

### Production of lipids

Lipids are organic compounds such as fats, waxes, oils, and steroids that are insoluble in water but soluble in organic solvents. Single-cell oils are produced commercially as dietary supplements in infant formulas using fungi such as *Mucor circinelloides* and *Mortierella alpina* [49, 50]. One such single-cell oil that is derived from fungus and used as a supplement for infant formula is the polyunsaturated omega-6 fatty acid Arachidonic acid (ARA), which is believed to be important in the development of the infant brain [51] . In addition to their widely-accepted use in making oils as infant nutrient supplements, microbially-produced polyunsaturated fatty acids are also being explored for use as biofuel precursor molecules [52, 53]. A biofuel is defined as a fuel derived from biomass, specifically living organisms and/or their metabolic byproducts, and are low-carbon alternatives to diesel. Production of biodiesel, a long-chain fatty acid ester-based biofuel, includes the following steps: cultivation, lipid extraction, and transesterification [54, 55]. Deriving biodiesel from lipids originating from filamentous fungi has advantages compared to biodiesel derived from plant- or microalgae-derived oils, as fungi have shorter growth cycles, are more agnostic to climate and season, and their cultivation can be more rapidly scaled. Unlike using plants to derive biodiesel, a fungi-based approach does not divert valuable food crops and is easier to harvest and extract than diesel derived from microalgae [56–58]. Industrial production of biodiesel from plant and food source oils uses lipases extracted yeast and fungi, which encourages further research to overcome the economic constraints of biodiesel produced by oleaginous microorganisms [59].

Biotechnological research on lipid-based biofuels focuses on three main goals: (1) enhanced lipid accumulation, (2) production from diverse carbon sources and (3) increasing efficiency and yield of extraction and transesterification.

#### Lipid accumulation

Lipid accumulation in biomass is typically achieved by creating a flux of carbon toward lipid synthesis. Omic tools are used to characterize metabolic differences between culture conditions and to identify genetic engineering targets for improved lipid production or accumulation. This reduces the number of culture conditions that need to be tried to optimize lipid accumulation. For example, Zhang et al. utilized transcriptomics to find that fatty acid synthesis pathway genes in the filamentous fungus *Glarea lozoyensis* were upregulated when the fungus used fructose as an alternative carbon source [60]. Their result indicated an increased amount of acetyl CoA resulting from upregulated ATP citrate lyase expression, and a lowered tricarboxylic acid (TCA) cycle resulting from downregulated citrate synthase expression, ultimately enhancing fatty acid synthesis and increasing the fatty acid yield by about 33% [60]. Multi-omic analyses of *Mortierella alpina*, a well-known oleaginous filamentous fungus which accumulates lipids under nitrogen limitation, suggest routes to regulate lipid synthesis [61]. Lu et al. showed that under nitrogen limitation the TCA cycle is blocked by the downregulation of isocitrate dehydrogenase, thereby diverting the carbon away from amino acid synthesis towards fatty acid biosynthesis [61]. Lu et al. further demonstrated that nitrogen-limited culture conditions elevated carbon redirection in *M. alpina* towards fatty acid precursor synthesis and recycling of existing carbon compounds into fatty acids [62]. Genetic interventions targeting AMP metabolism and TCA cycle repression consistently improve lipid accumulation in *M. alpina* by redirecting carbon flux towards fatty acid synthesis [63–65]. Future research should therefore focus on further repression of the TCA cycle, and other biological pathways that divert carbon away from the production of lipids.

#### Carbon source

In addition to increasing the amount of lipids produced by the fungus, another important goal is to enable the use of cheap and renewable carbon sources such as agricultural and other biowastes. Filamentous fungi can grow on a wide range of carbon sources and have a high tolerance to inhibitory side products. This latter advantage is particularly important in single-cell oil production using lignocellulosic and agro-waste substrates, where pre-treatment of these raw materials generates inhibitory compounds [57]. Subhash et al. used lignocellulosic (corncob waste liquor, paper mill effluent) and cellulosic bio-waste (de-oiled algae extract) as cheap carbon sources for fungal single-cell oil production using *Aspergillus awamori* [66]. The types of fatty acids that could be derived from each waste were slightly different. For example, the precursor to the 8-carbon caprylic acid methyl ester was produced by degrading all three waste sources, but the precursor to the 22-carbon erucic acid methyl ester was only produced from degradation of pulp mill effluent. The production of lipids was observed to be slightly higher with the de-oiled algae extract than with the other two wastes studied. Importantly, fatty acid methyl esters (FAME) analysis confirmed *A. awamori*-derived lipids have comparable characteristics to vegetable-derived biodiesels like soybean oil [66]. Bento et al. used sugarcane molasses for single-cell oil production using *Mucor circinelloides* URM 4182 [67]. The biodiesel produced by this fungus was found to meet international standards. There are other examples of filamentous fungal single-cell oil fermentations that have produced FAME comparable to alternative biological sources such as rapeseed oil, which is currently used in 80% of European-produced biodiesel [55, 56]. It is clear that use of alternate carbon sources can lead to commercially viable lipid production from fungi. A thorough techno-economic assessment (TEA) is beyond the scope of this review, but should be conducted to find which feedstock leads to the most economic production of lipids on large scale. The TEA of producing oils with fungi should be compared to a TEA already conducted for other biological production of oils from oleaginous yeast, such as the one done by Bonatsos and co-workers, which states that the estimated minimum selling price of single cell oils for commercial viability is $4–6/kg [68]. Any TEA conducted on production of products by filamentous fungi will likely vary from country to country, as the dominant byproducts of each country’s agricultural sector will be different, as well as the costs of labor and capital. Conducting a TEA may also reveal that it is more economically viable for a fungus to have slightly lower yields on a much cheaper substrate, rather than maximizing production using a relatively expensive substrate. The TEA would also reveal the circumstances in which use of oleaginous yeast would be favorable to the use of filamentous fungi. The expertise of the business community should be consulted to drive future research priorities in this space.

#### Cultivation and processing

**Fig. 3:**
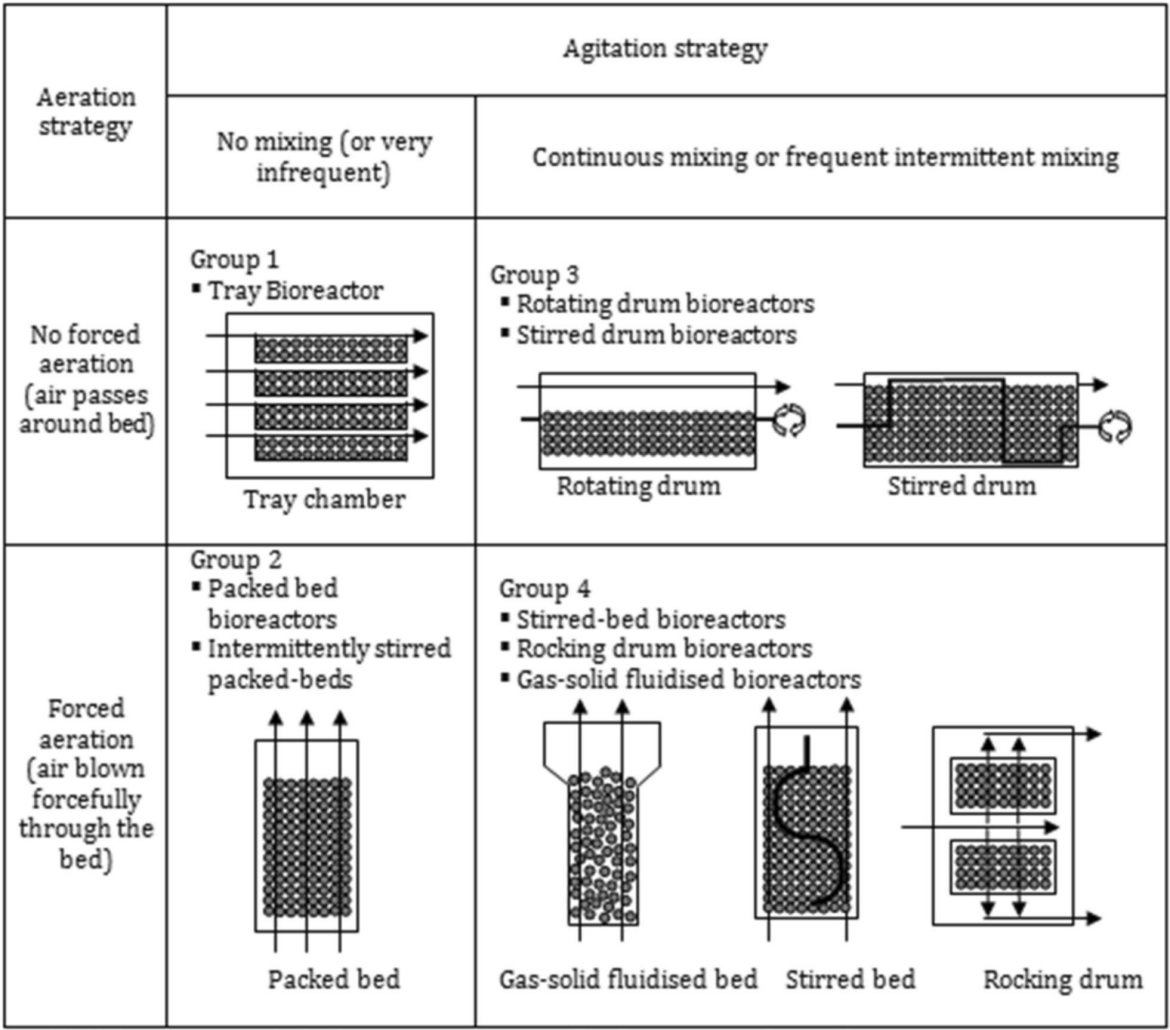
Types of solid-state bioreactors. This figure illustrates four types of solid state fermenters (SSF), depending on aeration and mixing. Figure is reproduced, unmodified, from the review of Manan and Webb [72], under a Creative Commons Non-Commercial Attribution License (CC-BY-NC 4.0)

Filamentous fungi can be cultivated using either liquid (submerged, SmF) or solid-state fermentation (SSF), with SSF often touted as cheaper than SmF. However, many factors influence the production and economic parameters, so choosing one over the other is complicated and few direct head-to-head comparisons are available. Zhuang et al. calculated that for cellulose production, solid state fermentation and submerged fermentation production costs were (in 2004 USD) $15.67/kg and $40.36/kg, respectively [69]. Mohanasrinivasan et al. demonstrated nearly twofold higher volumetric activity of lipase from three fungal genera when grown in solid-state [70]. Castilho and colleagues calculated that for a 100m_3_/yr production of lipase, SmF had 78% higher capital cost and the production cost was 68% higher than the product value, indicating no profitability for the SmF process. Total production costs were over threefold higher with SmF while payback time and ROI were 1.5 years and 68% for SSF [71]. The various types of solid-state bioreactors are reviewed by Manan and Webb [72], and their illustration of the various types of solid-state bioreactors is provided as Fig. 3.

Liquid-state cultivation has the benefit of homogenous medium allowing for even nutrient, heat, pH, and oxygen dispersion as compared to solid-state cultivation. However, liquid-state cultivation is costly because it requires high volumes of water and electricity for temperature control and constant stirring [73]. In contrast, solid-state cultivation requires less energy [74], and the limited amount of water present reduces risk of bacterial contamination and can provide higher product titers [75]. The low water content also concentrates enzymatic activity and promotes a substrate concentration gradient [76] while the use of agricultural and food processing wastes as substrates decreases costs [75]. Solid state fermentation more closely represents native growth conditions and promotes enzyme stability and decreased substrate inhibition, however, control over process parameters such as temperature, pH, and nutrient delivery are more difficult than in the case of submerged fermentation [75]. The choice of solid-state or liquid-state cultivation can have other phenotypic effects; RNA expression analysis revealed that chlamydospores produced via solid-state cultivation are potentially capable of synthesizing more fatty acids than hyphae grown in liquid-state cultures [73]. Fatty acid regulatory enzymes are differentially expressed under solid-state conditions in comparison to liquid-state, resulting in different fatty acid profiles and total fatty acid content [73].

While fungi are capable of producing products which are equivalent to fuels currently on the market, extraction and conversion of lipids produced by fungi remains laborious and costly, and this limits the market-competitiveness of these potential biofuels [77]. Single-cell oil extraction is typically completed by Soxhlet or Folch techniques with various modifications for cell disruption including microwave, ultrasound, and bead milling [57]. The extracted lipids are then converted to FAME via transesterification. Langester et al., demonstrated that the extracted fatty acid components vary by species and preferred cultivation conditions, regardless of extraction method, meaning that lipid extraction and transesterification have to be optimized for each species and cultivation method [77].

Despite advances in lipid accumulation in fungi fed with low-cost carbon sources, the downstream processing of filamentous fungal single-cell oil remains costly compared to traditional diesel [57]. Single-cell oil production from fungi is more feasible as a biorefinery concept in which costs are offset by the collection of other commodity metabolic products [57]. This suggests that future research should focus not only on genetic engineering for increased lipid accumulation, but also on advanced processing techniques to more easily separate the lipids from the fungal pulp. Genetic engineering could also be geared towards making economically-valuable bioproducts in addition to the lipids. The following section discusses some of the other secondary metabolites that are commonly made by fungi.

### Secondary metabolites

A secondary metabolite is typically a small molecule produced by an organism that is non-essential for growth. Secondary metabolites are produced by enzymes encoded by groups of genes co-located in a region of the genome. These enzyme groups are referred to as biosynthetic gene clusters (BGCs). Of the estimated 33,300 microbial secondary metabolites described to date, 15,600 metabolites are reported to have fungal origin [78]. Between 1993 and 2001, over 1500 compounds were isolated from fungi and over half of those compounds demonstrated anticancer or antimicrobial activity [79]. BGCs are highly diverse and vary considerably even within the same genus. Kjærbølling et al. reported that 15–27% of all identified genes in the 13 most closely related species of genus *Aspergillus* are not shared. In particular, *A. fumigatus* and *A. novofumigatus* share 24 proposed gene clusters out of 34 and 56 total predicted BGCs in their genomes, respectively [80]. This metabolic diversity within the fungal kingdom suggests a plethora of novel secondary metabolites remain undiscovered, some of which may be beneficial to human health or industry. In this section, we specifically discuss secondary metabolites with health implications, and those that are used as pigments and colorants.

#### Secondary metabolites with health implications

**Fig. 4:**
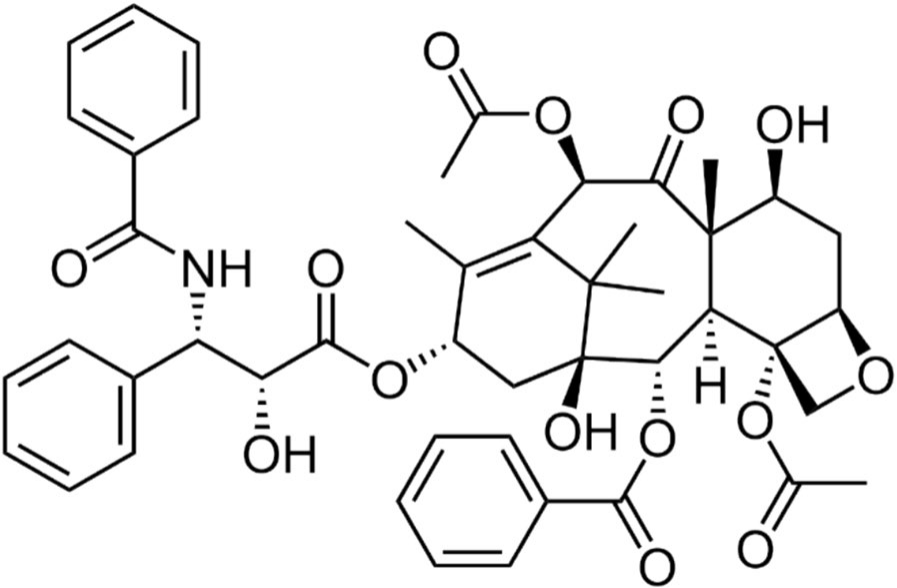
The chemical structure of Taxol. The complexity of the molecule makes it difficult to synthesize, and therefore fungal production of this molecule is attractive

Filamentous fungi-produced secondary metabolites can have important medical applications, such as the antibiotic penicillin and the anti-cancer Taxol (chemical name “paclitaxel”). Taxol is currently produced from the bark of wild-type yew trees using a costly extraction process with a low yield, with approximately ten tons of yew tree bark (from around 300 trees) being required to extract 1kg of Taxol, which serves approximately 500 patients [81–83]. Taxol is a very large and complicated molecule, meaning its total synthesis from petroleum products is not straightforward. While plants and microorganisms can be engineered to produce Taxol or its precursors, fungi can prove to be a feasible alternative to taxol production [84]. Taxol is produced in over twenty fungal species, including the endophytic fungi *A. niger* and *Fusarium oxysporum*, meaning that filamentous fungi may be an attractive alternative chassis for industrial Taxol production [82, 83, 85]. Recently, El-Sayed et al. published a review that noted limited improvements to Taxol production through random classic mutagenesis. The authors instead encourage the use of genome mining and bioinformatics tools in synthetic biology to expedite the process of scaling up from small- laboratory-scale to large industry-scale production of Taxols by fungi [86]. Taxol production has already been improved through focused genomic and transcriptomic studies of yeast. For example, studies have shown that improving the availability of the terpenoid precursor acetyl-CoA, via overexpression of pyruvate dehydrogenase, acetaldehyde dehydrogenase and acetyl-CoA synthetase, as well as overexpression of the Taxol pathway enzymes taxadiene synthase, hydroxyl-3-methylglutaryl-CoA reductase (HMG-CoA), and geranylgeranyl diphosphate (GGPP) synthase have resulted in enhanced product yields [87, 88]. After the Taxol is produced by fungi, it has to be extracted and purified, however, industrial-scale extraction protocols already exist for removing 1 kg of Taxol from 10 tons of yew tree bark (i.e. Taxol concentrations are 100 ppm). Even if yields of Taxol in fungi are small, processes can likely be adapted to extract it efficiently (Fig. 4).

**Fig. 5:**
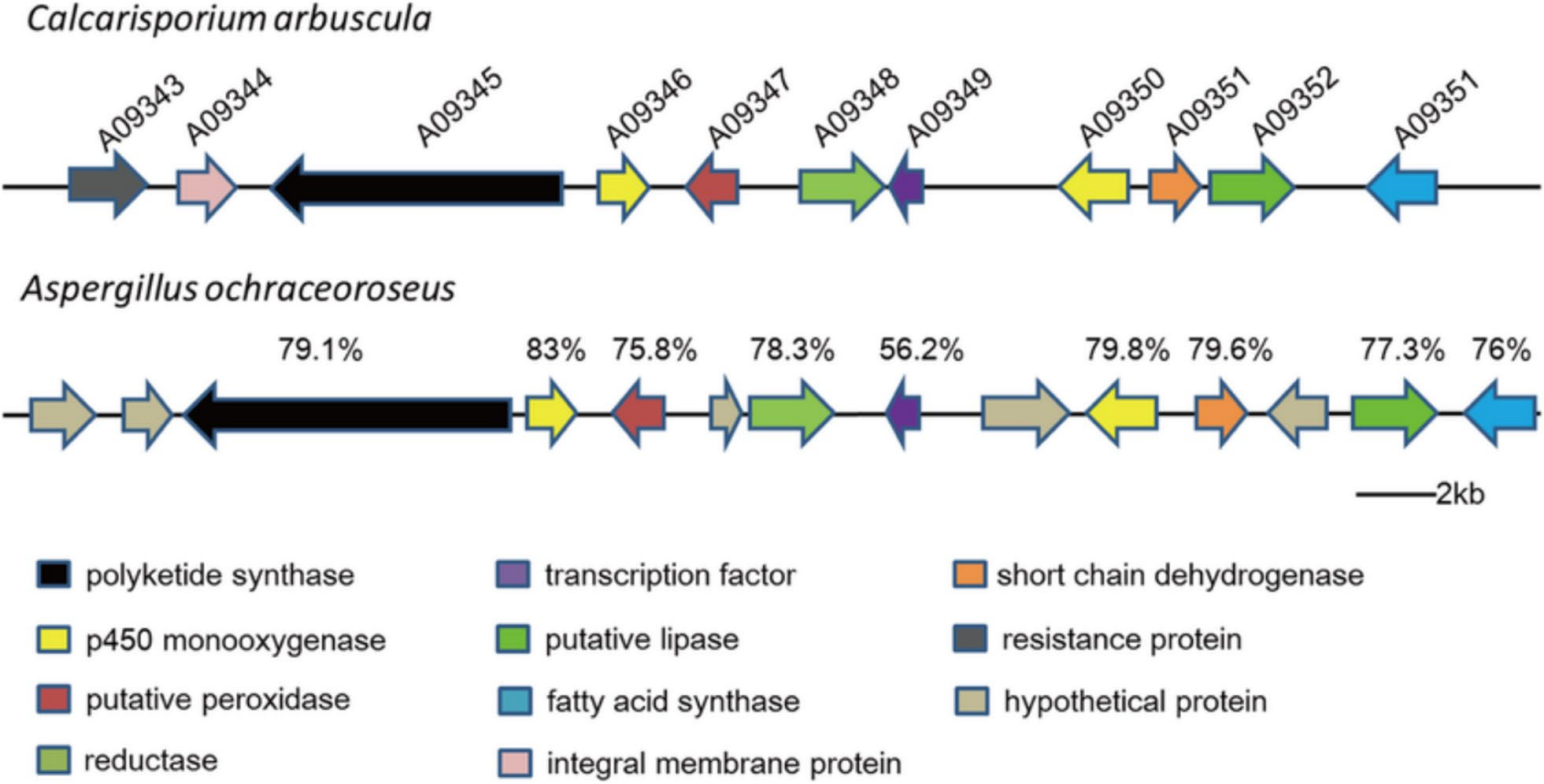
Two examples of aflatoxin gene clusters [95]. Figure is reproduced under a Creative Commons Attribution 4.0 International License Reproduced from the work of Cheng and co-workers

Not all secondary metabolites produced by fungi are beneficial. The Food and Agriculture Organization of the United Nations reported that 25% of global agricultural products are contaminated by mycotoxins yearly [89]. The number of known harmful metabolites is increasing with characterization of novel fungal genomes, revealing more targets of investigative interest [90]. The T-2 class of type A trichothecene mycotoxins produced by *Fusarium* and aflatoxins produced by *Aspergillus* species are of great concern to human health [91–94]. These aflatoxin gene clusters can be quite complex, as illustrated in Fig. 5 below from the work on Cheng and co-workers [95].

T-2 toxins are non-nitrogenous compounds that target eukaryotic protein synthesis. T-2 toxin is produced by 10 biosynthetic genes referred to as the Tri5 gene cluster, which is conserved across many filamentous fungal species [96]. T-2 toxin causes chronic acute toxicity and induces apoptosis in immune system cells [97].

Logistically, there are limits to food processing decontamination capabilities, especially as fungal growth is particularly difficult to address. Mycotoxins can be detected using a range of techniques such as thin-layer chromatography, LC/MS, HPLC, and ELISA-based methods, however, these are slow and tedious [98]. More rapid techniques include PCR, spectroscopy, and hyperspectral imaging [98]. The use of aptamers and enzyme-based biosensors have also been demonstrated [98]. Rapid and efficient detection of mycotoxins is important in the food industry, as these toxins can have serious health consequences if ingested [1, 99].

Due to the resilience of fungal contaminants and production of secondary metabolites such as mycotoxins, harsh physical and chemical methods are used to decontaminate food products [92, 100]. These physical and chemical methods, such as heating to 250 °C or treating with alkaline agents, damage food quality [100–102]. The food industry implements other control mechanisms to limit aflatoxin strains during storage and processing such as drying and sorting, irradiation, ozone and other chemical agents, and specific packaging materials [98, 103]. In the field, crop rotation, mold-resistant crop strains, pest control, water and nutrient management, and application of advantaged non-aflatoxin-producing fungi as competitors are all used to reduce fungal contamination are used as potential aflatoxin mitigating strategies. [98, 103].

Transcription factors (TF) play an important role in regulation of toxic secondary metabolites in fungi. Two pathway specific TFs, encoded by *aflR* and *aflJ*, located within the aflatoxin (AF)/sterigmatocystin (ST) gene cluster are involved in regulation of AF/ST gene expression in *A. parasiticus*, *A. flavus*, and *A. nidulans* [104, 105]. Deletion or mutation of either of these genes results in reduction in AF/ST gene expression and significant reduction of AF/ST production [106–109], while over-expression of these genes results in an increase in AF/ST production [110–112]. Regulatory controls imparted by transcription factors such as LaeA, AoKAP2, VeA, and BrlA are known to regulate secondary metabolism in fungi [104–113]. Secondary metabolite production pathways can be modulated by methods beyond direct gene editing by targeting epigenetic regulation [114]. For example, *HdaA* that codes for a histone deacetylase is shown to be involved in telomere-proximal secondary metabolite cluster suppression in *A. nidulans* [115]. Deletion of *HdaA* results in increased levels of sterigmatocystin (a mycotoxin), penicillin and terrequinone A in this fungus. Overexpression of histone acetyltransferase HAT1 that increases chromatin acetylation resulted in restoration of secondary metabolism in *Fusarium fujikuroi* [116]. This demonstrates that toxin and secondary metabolite production can be achieved by controlling expression of acetylases and deacetylases. Remediation of type A and B trichothecene mycotoxins such as T-2 triol and deoxynivalenol, respectively, has been demonstrated with *Eggerthella* sp. DII-9 (chicken intestine-isolated bacterium) via enhancement of de-epoxidation of trichothecenes [91]. Non-mycotoxin producing strains, both bacterial and fungal, can also be applied as competition to the mycotoxin producing strains as a mode of mycotoxin production reduction by growth limitation or mycotoxin degradation [91, 92]. For example, aflatoxin production and *Aspergillus flavus* growth is shown to be inhibited by culture filtrates of *Eurotium cristatum*, a probiotic fungal species isolated from Fuzhuan brick-tea [92]. A summary of the various control strategies used for mycotoxin decontamination is provided in Table 2.

**Table 2:** Control strategies for fungal mycotoxins: genetic, chemical, and process-level interventions

Secondary metabolite type	Control strategies	Reference
T-2	Physical methods	[101, 102]
	Chemical agents	[101, 102]
	Enzymatic/biological	[91]
Aflatoxin	Non-mycotoxin producing bacterial and fungal strains	[91, 92]
Ochratoxin A	Degrading enzymes	[100]
Aflatoxin	Epigenetic control	[117]
Secondary metabolite gene cluster	Transcription factor control (aflR, aflJ, Lae, AoKAP2, VeA, BrlA)	[104–109]
Sterigmatocystin, penicillin, terrequinoneA	Epigenetic control—HdaA	[114, 115]
Secondary metabolism	Epigenetic control—HAT1	[116]

Identifying and cataloging novel secondary metabolites creates a database which will inform screening and engineering efforts. Characterization of novel secondary metabolites is valuable to health in terms of identifying potential toxins as well as potential antimicrobial/anticancer compounds. Screening for biosynthetic gene clusters and control mechanisms often requires species-specific omics experiments, followed by either genetic engineering for enhanced production or process engineering for optimization of fermentation. Several key technologies need to be developed and integrated to realize the full potential of mining genomes for novel secondary metabolites: better annotated fungal genome databases, streamlined gene validation studies for genes of unknown function, systematic metabolome profiling, and mutant libraries.

#### Pigments and colorants

Mycopigments are a group of fungal secondary metabolites with tremendous industrial relevance and potential. Additionally, fungi exhibit rapid growth patterns which can be genetically manipulated for promoting higher pigment yields [118, 119]. Fungi can produce a plethora of pigments that have applications for food, feed, cosmetics, and as additives for material production. Filamentous fungi have been shown to produce β-carotene, melanins, azaphilones, quinones, flavins, ankaflavin, monascin, anthraquinone, and naphthoquinone thereby producing a spectrum of colors (yellow, orange, red, green, purple, brown, and blue) [118]. Pigments also have medical usages, such as pigments from Antarctic fungi with antimicrobial and antiparasitic activity [120], and in photodynamic therapy against cancers. Additionally, pigments are used in the food and textile industries, and ongoing research is investigating safe fungal-derived pigments for consumption to reduce population exposure to harsh synthetic pigments currently dominating the industry [121–123]. The field of mycopigments is very large, and several book chapters and literature reviews cover fungal pigments in detail [119, 122, 124]. Of particular note, Dufossé addresses recent literature covering several filamentous fungi of interest for pigment and colorant production for industrial use in a recent book chapter [125]. As of 2025, food grade pigments on the market include *Monascus* pigments, Arpink red from *Penicillium oxalicum*, riboflavin from *Ashbya gossypii*, and lycopene and β-carotene from *Blakeslea trispora* [125]. There is also a living reference work entry by Caro and co-workers that serves specifically as a collection of filamentous fungal pigment and colorant research for increased and improved biotechnological processes for industrial applications [126]. As there have already been extensive reviews elsewhere, we give only a brief overview here. Biochemically many fungal pigments fall into two categories: carotenoids and polyketides, as outlined below.

Carotenoids are terpenoids containing 40 carbon atoms in their structure. They vary in color from yellow to orange-red due to the presence of light-absorbing conjugated double bonds, and are known to protect cellular macromolecules against photo-oxidation [127]. Industrially relevant fungal carotenoids include β–Carotene, lycopene, canthaxanthin, astaxanthin, torulene, and torularhodin. Carotenoids are frequently used in the food sector for their powerful antioxidant properties [128]. Fungal polyketides are tetraketides and octaketides with eight C2 units that link together to create a polyketide chain. Naturally occurring polyketide pigments produced by fungi include melanins, anthraquinones, hydroxyanthraquinones, azaphilones, quinones and naphthoquinones [118, 129].

Additionally, fungi also synthesize riboflavin, a yellow, water-soluble vitamin that is commercially used as a food colorant. Commercially, riboflavin is predominantly generated by three microorganisms, the ascomycetes *Ashbya gossypii*, the yeast *Candida famata*, and the bacterial species *Bacillus subtilis *[130].

One of the key advantages of filamentous fungi-based pigment production is that many species can metabolize non-preferred carbon sources and persist in harsh conditions, including biowaste. In contrast, while many dyes can be derived from plant and animal sources, this diverts arable land away from food production. There are several examples of filamentous fungi grown on various biowastes for pigment extraction. For example, *Neurospora intermedia* produced 1.54 mg carotenoids/g during growth on baker’s yeast wastewater, while simultaneously reducing the chemical oxygen demand (COD) by over 30%, and producing additional value-added products [131]. Food waste is another feedstock of interest for filamentous fungi for production of pigments, with Troiano and co-workers using a co-culture of *Trichoderma reesei* and *Talaromyces albobiverticillius* on a simulated food substrate to generate pigments [132]. Lignocellulosic wastes as substrates for pigment production is also extensively explored, and is reviewed elsewhere [133]. There remains a large barrier to commercialization of particularly food products from waste streams as rigorous toxicity and allergen criteria must be met according to regulatory requirements. Consumer acceptance would also be a formidable barrier to marketability.

Efforts to improve extraction of pigments have been reported as in Wei et al. who detail an approach to extracting green pigment from *Metarhizium flavoviride* spores [134]. Ultrasound-, pressurized liquid-, microwave-, supercritical CO_2_-, ionic liquid-, and pulsed electric field- assisted techniques have been considered for extraction of pigments [135]. However, there is a huge challenge in trying to scale any of these extraction strategies, which is the final remaining hurdle to scaled up pigment extraction from filamentous fungi.

Production of endogenous fungal secondary metabolites such as pigments in heterologous organisms such as yeast can be ineffective, encumbered by toxin byproducts, or limited to specific growth conditions that aren’t yet amenable to large scale fermentations. In comparison, Ree Yoon et al. successfully increased yellow, orange, and red pigment production in the filamentous fungus *Monascus ruber* via CRISPR-mediated mutagenesis of negative regulators of its pigment biosynthesis pathway [136]. One of the challenges in pigment production in *Monascus* strains is the co-production of toxins, such as citrinin, which is induced in a few of the CRISPR-generated mutants. Increased pigment production can also be mediated non-genetically via community engineering. Co-culturing of *Monascus* with *Lactobacillus fermentum, S. cerevisiae,* and *A. oryzae* have led to increased pigment production [137, 138]. Similar increases in pigment production have also been observed in *C. neoformans* when co-cultured with *Klebsiella aerogenes.* However, co-cultures are not currently being used at industrial scales, and the benefits of cocultures have yet to be demonstrated at pilot plant or industrial scales. More research is needed on community dynamics before co-cultures can be brought into product development.

Mycopigments offer immense benefits over other natural pigment sources and synthetic counterparts, including safety, eco-friendliness, and the ability to survive harsh conditions, while using waste biomass as substrates. Some fungal pigments are already produced at industrial scales and some are on the commercial market as food additives, but more research is needed to drive down economic process costs, improve toxicity screening for food-based products, and bring new promising products to the marketable scales.

### Enzymes for plant biomass degradation

Using lignocellulosic biomass as industrial fungal fermentation feedstocks can lead to numerous environmental benefits. These benefits include displacing fossil fuel use, capture of CO_2_ through photosynthesis, carbon diversion out of landfills (and subsequent greenhouse gas GHG emissions), and potential carbon sequestration in biomaterials. However, economic production of biomolecules and biomaterials, especially at commodity scale, requires minimized production costs. While lignocellulosic biomass, especially from agricultural or other waste material, can be inexpensive, there are still costs associated with transport, processing, pretreating, hydrolyzing, fermenting, and recovering the product. Arguably, the enzyme hydrolysis is the most expensive and crucial step to releasing the sugars in this process [139]. This requires inexpensive, high activity enzymes to minimize production cost of sugars and other compounds for use as feedstock in industrial fermentations.

A wide range of enzymes are currently made on an industrial scale. Industrial fungal enzymes play major roles in starch hydrolysis and deconstruction of structural polysaccharides in plant cell walls, specifically cellulose and various hemicelluloses. As plants comprise around 80% of the biomass-based carbon on the planet [140], it is unsurprising that fungi have evolved a highly complex and adaptable suite of enzymes targeting this vast sugar reservoir [141] and the vast majority of industrial biomass-degrading enzymes are produced by filamentous fungi. Cellulases, amylases, pectinases, lytic polysaccharide mono-oxygenases, proteases, cutinases, xylanases, other hemicellulases, various esterases, glucuronidases, inulinases, xyloglucanases, and many other carbohydrate-active enzymes are all produced and secreted by numerous filamentous fungi. An entire classification system, the CAZy.org database, has been developed to keep track of these enzymes across all kingdoms of life.

The CAZy database organizes carbohydrate active enzymes (CAZymes) into clans based on structural fold and families based on activity. A study by Lange et. al., used a novel peptide pattern approach to identify carbohydrate active enzymes (CAZyme) “hotspots” among un-annotated fungal genome data and assign both “function” and “family” to each predicted protein [142]. The number of “Function;Family” observations within a genome is an indicator of CAZyme potential activity and diversity and was used to rank the 1932 strains in the study for potential overall activity. Perhaps most surprising is that none of the most prevalent and most well-studied fungal workhorse strains make the top 10 list in any substrate category of CAZymes, even when adjusted for redundancy. This suggests that there is a richness of CAZymes in under-studied species, highlighting the need for further research on these organisms.

Proteins produced by filamentous fungi play an important role in industry, such as cellulases produced by *Trichoderma reesei* for sugar liberation from lignocellulosic biomass for subsequent conversion to alcoholic biofuel [54]. Exerting control over protein production in filamentous fungi via genome engineering affords large advances in protein characterization, expression, and secretion. Wang et al. published a review describing various ways to engineer protein production pathways to successfully secrete target proteins, which will not be reviewed here [143].

#### Cellulases and hemicellulases

Numerous reviews have been published over the past several decades describing the activity and interplay of the various cellulases and hemicellulases in degrading plant cell walls [144–153]. An entire issue of Fungal Genetics and Biology was devoted to the degradation of plant cell walls by fungal enzymes [141]. It is critical to understand that plant cell wall components are complex and interconnected, meaning a complex set of enzyme activities is required to facilitate their deconstruction. The β-1→4 glucose polymers that make up cellulose are arranged in aggregates that form fibrils, with bundles of 36 chains making up the most common unit [154]. The highly diverse amorphous hemicelluloses provide a hydrophilic matrix to contain and bundle together cellulose fibrils. Hydrogen bonding of hemicellulose backbones to cellulose provides a strong interconnection between cellulose fibrils, promoting the strength and rigidity of the cell wall. The most common hemicellulose, xylan, is comprised of a decorated β-1→4 linked xylose backbone. The sidechains vary significantly depending on the species and tissue type, but function to retain water and cross-link to the third major plant cell wall polymer, lignin [155, 156].

Cellulases are divided into three basic categories: endo-, exo-, and oligo-acting hydrolases. Cellulases work synergistically, with exocellulases removing glucose or cellobiose from the cellulose chain ends, endocellulases primarily acting to open additional chain ends by cleaving in the middle of the cellulose molecule, and β-glucosidases (oligomerases) hydrolyzing short soluble cellodextrins to glucose [153]. Fungal cellulase systems are heavily reliant on the exocellulases for the great majority of the depolymerization. These enzymes, mainly glycoside hydrolase family 7 cellobiohydrolases (GH7 CBHs), are not found in prokaryotes. Fungal GH7 CBHs have a multidomain structure heavily dependent on disulfide bonds and glycosylation (N- and O-types) to impart both structural stability and activity, and are very difficult to express in prokaryotic and most non-fungal eukaryotic systems due to these factors [157].

For many years, the production of alcohol biofuels such as ethanol or butanol from cellulosic feedstocks promised to transform our liquid transportation fuel infrastructure and vault cellulases to the top of the industrial enzyme market. Major industrial enzyme producers such as Novozymes, Genencor/Danisco/DuPont, DSM, Iogen, and others developed highly engineered cellulase and hemicellulase formulations targeted to specific lignocellulosic biomass feedstocks. Cellulase and hemicellulase production was heavily researched during the late 1990s and 2000s, primarily driven by the U.S. Department of Energy’s interest in lignocellulosic ethanol. Both Novozymes A/S and Genencor International leveraged federal funds totaling $32M to develop a series of improved enzyme for biomass hydrolysis [158]. Novozymes released their Cellic® Ctec/Htec series while Genencor marketed their Accellerase® series [139]. Despite significant improvements in activity and reduction in production costs through the mid 2010’s, the failure of multiple lignocellulosic ethanol demonstration plants and the resultant lack of market development essentially stopped further industrial research [159]. Despite this major failure, cellulases continue to be the 3_rd_ most prevalent industrial enzyme, comprising about 15% of the global enzyme market [150, 160]. Fungal cellulases are used in textile manufacturing to polish cotton cloth and “stone wash” denim, in biopulping and biobleaching applications for pulp and paper production, de-inking in paper recycling, improving quality in various food and beverage applications, and in upgrading animal feed [161]. In brewing, cellulases are used to improve the yield and drainage of barley wort. Cellulases are used synergistically with hemicellulases and pectinases (collectively termed macerases) to clarify wine and fruit juice, increase juice extraction, and decrease juice viscosity [153]. Similar effects are apparent in the extraction of olive oil, pigments and other bioactive compounds with these same enzymes.

For decades, researchers have attempted to increase cellulase activity. Initial development work at the US Army Natick Development Center, Rutgers University, and other labs focused on random mutagenesis of the fungus, generating a breadth of high- and hyper-producing strains of *T. reesei*, the most commonly known being QM9414 and RutC30 [162–165]. A huge amount of effort was expended (and continues to be applied) in increasing cellulase production through strain engineering, mutagenesis, culture growth and induction optimization, and other methods for *Trichoderma* and other species [166–184]. As molecular biology expanded to fungal systems, researchers looked to improve the catalytic activity of these industrial enzymes. This presented a much different challenge, as the complex and synergistic nature of the cellulase system made it difficult to attribute improved activity to a single enzyme. Increasing thermal stability and pH tolerance were relatively easy targets for the endoglucanase enzymes and several groups were successful in engineering these traits into multiple endocellulases [185–193]. In contrast, engineering catalytic activity enhancement has proven difficult, though a couple of successful examples have been reported [194, 195].

Engineering of fungal exocellulases or cellobiohydrolases has proven even more difficult, mainly due to the difficulty in finding a suitable expression host [196]. The domain complexity, glycosylation requirements, and disulfide bond network requirements impose severe challenges on suitable expression hosts. It can be difficult to conduct accurate assays to test enzyme activity because the substrates are insoluble, multiple enzymes are acting synergistically, the assays require long run times, and the biochemistry of the substrates are variable (i.e., raw biomass, pretreated biomass, cellulose powders, etc.). In addition, it can be very difficult to accurately compare the results of enzyme assays produced by different labs. The results of assays are reported inconsistently across the literature. Activity is reported across many different substrate analogues, such as various cellulose preparations and dyed polysaccharides. Even when the same substrate is used, the particle sizes may be different and this is a major factor in enzyme accessibility and activity. The literature also reports different colorimetric or fluorometric small molecule assays, rendering direct comparison of results across multiple labs difficult. Activity units reported are inconsistent as well, being variably reported on a mass protein per mass of biomass, mass protein per mass glucan, or volumetric basis. The classic filter paper unit assay is still used, despite measuring only 3.6% conversion of a pure cellulose substrate. Even protein quantitation is inconsistent as different assays are often used. Enzyme activity measurement conditions are also inconsistent, as some report initial rates while others use time to target conversion extent or conversion at a designated time. This is highly confounding as meaningful levels of hydrolysis often takes days. There is clearly a need for better standardization of cellulase activity assays. At a minimum, assays should be carried out on a mass loading basis of mass protein per mass substrate, either on a glucan content or total mass basis. If total mass is used, the glucan content must be provided. Additionally, assays should target 80% conversion of the glucan or a minimum hydrolysis of 3 days. While standardizing the protein quantitation method would be difficult, glucan content in the majority of lignocellulosic substrates has been standardized in the National Renewable Energy Laboratory's (NREL) laboratory analytical procedure (LAP), which is generally recognized as the standard method for biomass analysis [197].

Despite this, several labs have managed to express and improve the activity of these proteins using native host expression and have conducted highly detailed activity assays to evaluate the results. Taylor et al. at NREL engineered biomass-targeted specific activity enhancement of *T. reesei* cellobiohydrolase I using domain swapping from *Penicillium funiculosum* Cel7A [196]. In follow on work, Brunecky et al. modeled hot spots of higher activity cellobiohydrolases across Cel7A natural diversity and engineered and tested those mutations in *P. funiculosum* Cel7A, increasing its native activity over 40%, which was 55% higher than *T. reese*i Cel7A [198]. Dotsenko et al. used rational design to engineering thermal stability into *Penicillium verruculosum* Cel7A, however the assays were 1 h on microcrystalline cellulose [199]. Building on this work, Pramanik et al. used in silico design to engineer *P. verruculosum* for increased thermal and ionic tolerance and demonstrated improved activities on aspen wood. However, results were reported in sugar concentration, not theoretical yield so it is unclear if there is a practical application for these variants [200]. Kolaczkowski et al. demonstrated increased activity of *T. reesei* Cel7A on microcrystalline cellulose through removal of N-linked glycans [201]. Another route to enhanced *T. reesei* Cel7A thermal stability utilized directed evolution [202] while alkali tolerance was engineered by mapping targeted hot spots from alkaline-tolerant cellobiohydrolase onto *T. reesei* Cel7A [203].

#### Lignin modifying enzymes (LMEs)

Lignin is a complex polymer of several phenolic- and non-phenolic-based compounds. Lignin is found in between cellulose-hemicellulose bundles in wood and reinforces the cell wall rigidity through its covalent crosslinks to xylan sidechains. Lignin’s hydrophobicity channels water through the vascular system of the plant and its phenolic nature provides durability and disease resistance. Fungal degradation of lignin is generally reserved for the “white-rot” Basidiomycetes, so called because the degraded wood appears white after the lignin is oxidized by laccases and peroxidases produced by the fungus [204–206]. Industrially, laccases are used to delignify wood to reduce pulping energy and bleaching chemical use, clarify juices and wine, and decolorize dyes and industrial waste effluents [207]. “Brown-rot” Basidiomycete fungi have limited activity on lignin and it is thought that this activity serves mainly to detoxify the lignin or to allow easier access to the cellulose [204, 208]. Similar to the brown-rot fungi, “soft-rot” Ascomycetes rely primarily on cellulases to degrade wood and produce limited LMEs to enhance this activity [204, 206].

Laccases, manganese peroxidases, lignin peroxidases, and additional peroxidases (versatile- and dyP-type) are referred to as LMEs, though the term “ligninase” is still used as well [205]. These LMEs are oxidative, not hydrolytic, and so their activity is somewhat promiscuous and their mode of action varies. Depending on the substrate and local redox environment, LME activity may result in depolymerization, ring cleavage, or polymerization. While direct oxidation of lignin is possible, an alternative path of oxidizing a soluble mediator is often observed. These mediators mainly take the form of metal ions, mainly Cu, Mn, and Fe, though organic acids, carbohydrate-derived compounds, alcohols, and other metabolites are also involved [209]. This system may be more effective at oxidation of complexed lignins which are not physically available to the enzyme. The net result is that this class of enzymes is capable of degrading not only lignin, but many other aromatic or phenolic-based compounds, resulting in applications in dye decolorization, environmental remediation, and industrial waste treatment in addition to delignification and bleaching operations in the pulp and paper industry [160, 210–213]. While lignin peroxidase (LiP) catalyzed oxidation of veratryl alcohol has been well-described as a mechanism to breakdown lignin or a range of other compounds, at least one study has suggested that a secondary system may be responsible for this activity [214–217]. Several strategies to enhance production and/or activity of the enzymes have been implemented to this class of enzymes, which include targeted enzyme engineering, heterologous expression, genetic engineering of the organism which expresses the enzyme, and growth parameter optimizations to improve native enzyme expression levels [218]. Omics studies have also contributed to improving the knowledge base of these enzymes with respect to their expression and regulation. While comparative genomics have been used as a tool for identifying genes and pathways inherent to the different fungal species [219, 220], transcriptomic studies have revealed crucial information on expression and regulation of lignin degrading enzymes in fungi such as *Tremetes versicolor*, *Phanerochaete carnosa*, *Phanerochaete sordida*, *Thielavia terrestris,* and anaerobic gut bacteria [221–225]. Some of the information revealed by the studies include the discovery of novel CAZymes, peroxidases and cellobiohydrolases that act against cellulose and lignin, and which are therefore targets for enzymology studies. While regulation of ligninolytic genes is still poorly understood, there are several instances where transcriptional induction of lignin degrading genes has been implicated in degradation of lignocellulosic substrates in white rot fungi (WRF) [226–228]. Some recent studies include comparison of *Phanerochaete* species where higher upregulation of ligninolytic enzymes in *P. sordida* YK-624 was observed under ligninolytic conditions compared to the model WRF of *P. chrysosporium* [224]. This supports *P. sordida* YK-624 having superior lignin degrading ability. Chi and Zhang observed induction of lignin degrading genes in response to woody environment in *Lenzites gibbosa*, suggesting their direct involvement in wood degradation [229]. Comparative transcriptomic studies combined with genomics have provided evidence for increased oxidoreductase potential, owing to the presence and expression of several MnPs and desaturases, and decreased cellulolytic activity owing to reduced expression of cellulolytic systems in *Ceriporiopsis subvermispora* in comparison to the model WRF, *P. chrysosporium *[219].

On the other hand, proteomics and metabolomics has helped provide evidence for translational effects of the expressed enzymes towards breakdown of lignocellulosic substrates [230–234]. A good review of lignin degrading enzymes has been published by Makela et al. [206].

#### Lytic polysaccharide monooxygenases (LPMOs)

Another recently discovered class of mono-copper enzymes, called the lytic polysaccharide monooxygenases (LPMOs), can also act on plant cell wall polysaccharides. They are classified as auxiliary active enzymes and have demonstrated to be active on a range of polysaccharides, including chitin, starch, xylan, and others [235–238]. Currently assigned to the Auxiliary Activity Family 9, the precise nature and activity mechanisms of LPMOs are still under investigation. What is known is that they can significantly enhance the activity of polysaccharide degradation, including cellulose, when combined with glycoside hydrolase enzymes and the proper redox cofactors. Studies describing efforts to enhance activity of these enzymes have started to emerge [239–242].

#### Pectinases

The fourth and final major plant cell wall polymer, pectin, is comprised of several acidic polysaccharides that function as a reversible glue to hold cells and tissues together. The primary backbone sugars are galacturonic acid and/or rhamnose (depending on the type of pectin), and several other sugars serve as sidechains, including galactose, arabinose, xylose and apiose. Pectins are not as complex or diverse as hemicelluloses, but their complete deconstruction still requires multiple enzyme activities. Pectin lyase (pectolyase) cleaves the α-1→4 linkage of methylated galacturonans in the pectin backbone, generating pectic oligosaccharides. Its partner, polygalacturonase, acts on linkages connecting non-methylated galacturonans. The methyl-ester on the galacturonan is cleaved by pectin esterase. Industrially, pectinases are primarily used to clarify and improve the flavor profile of wine and fruit juices. Additional applications include enhancing juice extraction from fruit, oil extraction from citrus peels and vegetables, and assisting in retting of plant material for fiber generation [243]. Several factors are known to influence production of pectinases in fungi, which includes medium components such carbon, nitrogen and minerals, as well as substrate and the cultivation mode [244–248]. Inducers also play an important role in pectinase production. Particularly, pectin, polygalacturonic acid, and citrus pulp pellets have shown to induce production of this enzyme family [249, 250]. Omics analyses have revealed crucial insights into the regulation and expression of these enzymes [245, 251, 252]. While culture parameters have shown to be critical in controlling pectinase production, important genetic features such as the F-box protein exo-1 and GAA1 have also been implicated in regulation of their expression [253, 254]. Targeted engineering of genomes is therefore an alternate route for enhancing pectinase production in fungi. Production and improvement of enzyme production have been reviewed intensively in the literature [243, 245–247, 249, 255–258].

### Other industrial enzymes

Fungal production of enzymes encompasses a wide range of applications and is a multi-billion-dollar industry [259]. Numerous industrial sectors rely on fungal enzymes to produce their products economically and sustainably. Historically, application of fungal enzymes to industrial processes has been carried out empirically, with process improvements relying on new enzymes or selectively pressuring the production strains. More recently, modern genetic tools and rapid genome sequencing have provided an unprecedented opportunity to advance these operations at a rapid pace. New activities can be introduced while production levels can be increased and undesired proteins can be deleted [260]. An excellent recent review on the potential of these techniques to improve industrial fungal enzyme production was recently published by Kun et al. [261]. Improving the yield of fungal production of enzymes has the potential to revolutionize industry, which already extensively uses these types of enzymes [158]. Proteases, amylases, cutinases, and lipases are used in food processing and detergents. Pectinases, amylases, and polygalacturonases are used in juice extraction and clarification. Amylases and glucoamylases are heavily utilized in brewing, baking, and sugar production. Invertase is used to produce high fructose corn syrup from hydrolyzed starch. Phytase, glucanase, amylase, cutinase, xylanase, and polygalacturonase are used in animal feed to improve nutritional quality. The textile industry uses a wide range of enzymes mentioned above to treat wool, soften cotton, process denim, and improve dye application, while lipases, amylases, and proteases dehair and process leather.

Proteases have significant advantages for industrial processes, tending to have high stability and retaining activity across wide pH, temperature, and solvent concentration ranges. Proteases are used to tenderize meats, in cheese-making, tanning of animal hides, and in detergents. Proteases are produced under submerged and solid-state fermentation conditions and regulated by the availability of carbon, nitrogen and sulfur, type of substrate, as well as end products such as amino acids and NH_4_^+^ [262, 263]. Detailed review articles discussing proteases are available elsewhere [264, 265].

With stability to pH and temperature ranges similar to proteases, lipases are used to modify the fat content of milk, cheese, fish, and other foods as well as in detergents. Other applications of lipases include biodiesel processing, treatment of sewage to improve flow and conversion, pharmaceutical and fine chemical manufacturing, flavor compounds, and modification of various edible fats and oils [266]. Production and activity of lipases are affected by various factors such as substrate type, temperature, pH, presence of metal ions and surfactants [267, 268]. Genetic engineering to enhance native and heterologous lipase production in fungi has been reported [269–271], yet there remains a huge opportunity for expanding synthetic biology towards improving the productivity of these enzymes in fungi.

Phytase cleaves phosphate groups from phytate (myo-inositol hexakisphosphate), a phosphorous storage compound found in many plant seeds. Phytase is used in the treatment of agricultural feed to increase phosphate availability and reduce phosphate levels in pig and poultry manure to mitigate the release of large amounts of phosphate in agricultural runoff. This removal of phosphate is done in order to prevent harmful eutrophication of aquatic ecosystems; eutrophication leads to algal blooms that can release toxins or severely deplete dissolved oxygen, killing local organisms [272].

## Fungi for sustainability

Enzyme production by filamentous fungi is a well-established industry with numerous applications, but fungi have more to offer. Their ubiquitous ability to breakdown complex natural and man-made polymers coupled to their genetic tractability, high productivity, and broad bioproduct capabilities is ready-made for exploitation in a sustainable biobased economy. A circular economy, whereby resources are used sparingly, and wastes are recycled back into useful products, is a major worldwide goal. Fungal biotechnology is an attractive component in growing sustainable economies due to several potential attributes: fungi are themselves biodegradable, and fungi can convert wastes into valuable products. As fungi can adapt to different climates, less-stringent environmental control (e.g., temperature, pH) is required, representing a savings in energy. It is estimated that >90% of existing fungal species have yet to be identified [273]. With a conservative estimate of ~5 million existing fungal species and the current trend of ~2000 new species identified every year [273], it will be far beyond our lifetime before they are all identified. This represents a vast untapped resource of strains to expand biotechnology capabilities in bioconversion processes. Newly identified strains may have the ability to use complex carbon sources of interest, or to be adapted to new processes such as fermentation. Bio-based products provide an opportunity to design biodegradable materials and reduce the reliance on petroleum-based products. Wood is the most common biological building material, and it also acts as a carbon sink. While wood is a renewable resource, it is in limited supply, so the ability to recycle wood products is increasingly important. Fungi naturally break down lignocellulosic biomass and have the potential to build novel products out of wood scraps, sawdust, worn out components, or woody composts that are not suitable for construction use [2, 274]. As discussed in Sect. 1.4, the breakdown of lignocellulosic material is perhaps the best studied aspect of fungal based bioconversion. This makes sense as fungi naturally inhabit forest floors and survive by their ability to break down complex carbon sources. The pulp and paper industry produces a variety of organic and inorganic by-products depending on the pulping process, including large amounts of cellulosic waste which may be useful as fungal feedstocks [275]. In a study by Asadollazedah et al., *Aspergillus oryzae, Mucor indicus,* and *Rhizopus oryzae* were assessed for conversion of pulp waste to fungal biomass protein. This is the first study demonstrating that both types of neutral sulfite semi-chemical (NSSC) pulping process by-products (spent sulfite liquor and spent NSSC liquor) can be used as feedstock in fermentation by fungi, and this fermentation can generate a mass with a crude protein content comparable to soybean meal [276]. Other organic waste streams that have been used in fungal biomass protein production include: wood refinery waste [277], winery biomass waste [278], fruit [279], wastewater [280–282], vinasse [283, 284], stillage [285, 286], rice polishings [287] and corn cobs [288]. While these types of waste streams have not been applied to the consumer market fungal biomass protein products, they represent an intriguing avenue for bioconversion to value-added products. In this section, we will discuss the use of fungi for the making of materials, and food products, from a variety of feedstocks.

### Mycelia-based materials

Aside from what they can produce, the biomass of fungi themselves has been explored for use in building materials and fabrics. Mycelia-based materials offer sustainable alternatives to textiles, timber, foam, and insulation, and with the benefit of low energy conversion of waste material into value-added products, mycelia materials fit well into green construction and consumer products paradigms. The wide variety of suitable inexpensive and available substrates, growth conditions, and material extraction/harvesting methods means that the physical and mechanical properties of mycelia materials can be tuned, which provides a spectrum of materials for construction and architectural applications. Certain mycelia-based materials are already being commercialized, but others exist primarily as interesting ideas that have yet to be proven. Much more research and development are needed to bring myco-materials to general use, however the drive for circular economies, and sustainable and biodegradable materials, means that there is increasing interest in this space. Most patented technologies are applied to the areas of packaging, automotive design, electrical circuit boards, textiles, or other composites [289]. Overall, there is great potential for mycelia materials to replace current technologies, and the commercialization potential for mycelium-based composites is the subject of a recent review [290]. This field is rapidly developing, and the quick commercialization and public acceptance of these materials mean that some of the most-exciting innovations in this field are probably not being published in the open literature.

Most of the research to date has focused on downstream processing, rather than genetic manipulation of the organism itself. This may stem from a fundamental problem of materials science: there aren’t many high-throughput screens available for vital materials properties such as Young’s modulus, compressive modulus, molecular weight, yield strength, or viscosity, among others. However, synthetic biology works best in environments where high-throughput screening is possible, and therefore it is tempting to use synthetic biology merely to improve the yield, which is relatively easy to measure in high throughput. However, there are likely avenues for improvement of materials properties using synthetic biology that remain unexplored, meaning that high-throughput methods for measuring materials properties should be developed.

#### Leather-like fabrics

The use of fungal biomass for fabrics originates from early recognition of the structural similarity between cellulose and chitin, the main component of hyphal cell walls. The realization that cellulose has structural similarity to chitin led to the use of fungal biomass-based materials in papermaking and wound dressings. The increased commercial and academic interest in mycelia is correlated with the rise in the number of biotech companies utilizing fungal mycelium to produce materials [289, 291, 292], such as MycoTech, Mogu SRL, MycoWorks, Ecovative Design, and BoltThreads. Some of these companies have released prototype products made from the fungal biomass-derived leather-like material, and these products are expected to soon be commercially available.

Imitation, faux, and artificial leathers are materials that look and feel like genuine leather made from the hide or skin of animals, but may not have mechanical and material properties identical to genuine leather. Synthetic or plastic leather (pleather) can be made from natural or synthetic microfibers coated in polymer, typically polyurethane or polyvinylchloride. Recently, eco-friendly leather has become a class of faux leather that appeals to the ethically and environmentally conscious. These eco-friendly leathers may be plant-based (companies include: Will’s Vegan Store, Pinatex, Desserto, Veja, Fleather, and Veerah), produced from engineered yeast (Zoa by Modern Meadow), from bacteria [293] (ScobyTec), or from fungi.

Some of the challenges facing the production of fungal leather-like material include material processing and growth of biomass. The primary chemical treatments of mycelia materials include: the addition of glycerol or sorbitol to increase water content; sodium hydroxide, acetic acid, or alcohol treatment to remove extracellular matrix components, denature proteins, bleach and deodorize the mycelia material and deacetylate chitin for crosslinking later; and lastly, crosslinking with agents such as genipin, adipic acid, or phenolics to improve tensile strength, tear strength, and abrasion resistance [292].

The material properties of fungal-based leather have some known drawbacks. Dimensional stability, flexibility, and resistance to degradation are known issues [294]. Much of the current research on leather-like material has focused on downstream processing of the material rather than on genetic manipulation of the strains that make the material. This is an overlooked area of opportunity; recent work by Appels et al., demonstrated that a single gene deletion in the mushroom-forming fungus *Schizophyllum commune* resulted in measurable changes in the mechanical properties of the mycelium tissue, suggesting that manipulations of other parts of the fungal genome may also have implications for materials properties. The deletion of the SC3 hydrophobin, which plays roles in cell wall composition and hyphae hydrophobicity [295–299], in combination with judicious selection of environmental conditions, resulted in the density of the mycelium increasing by a factor of ~2 depending on growth conditions (e.g. from 0.8 to 2.1 g/cm^3^ under one set of growth conditions) along with a threefold–fourfold increase of the Young’s modulus and tensile strength of the engineered strain mycelia compared to wild type [300].

The longevity of genuine leather products is part of their appeal, and so the lifespan of fungal leather-like material products will be heavily scrutinized. More thorough studies are needed to investigate the growth conditions most amenable to uniform color, thickness, and consistency of mycelial tissue. The major bottleneck would be in the material properties testing, where throughput is currently limited. However, given the success in driving industrial scale production of cellulose from bacteria [301], there is reasonable precedent to accomplish a similar drive for fungal leather-like materials. More research is needed to investigate the use of additives in material processing, as using certain processing chemicals may reduce the biodegradability and sustainability that make fungal leather-like materials so appealing.

#### Biocomposites for packaging

There is an enormous demand for packaging materials. Eurostat estimates that the average inhabitant of the European Union generates over 180 kg of packaging waste per year, with 80 kg of that waste being paper and cardboard (https://ec.europa.eu/eurostat/statistics-explained/index.php?title=Packaging_waste_statistics). The ability to grow single-use packaging material that would naturally degrade would therefore represent a potential advancement in sustainability. Mycelium-based composites are grown by leveraging the natural ability of the fungi to infiltrate lignocellulosic material with a mycelia network. Typically, waste material such as sawdust is hydrated, macerated, sterilized, placed in a mold and then inoculated with fungal spores, fruiting bodies, or growing mycelia. After a period of days to months, the molded product is hot pressed or dried, thereby inactivating the fungi. Depending on the process, the bricks can be used as structural or insulative materials [302]. There are several excellent reviews covering various aspects of the life cycle of mycelia building materials, from biomanufacturing to application [2, 291, 303–306]. More details on mycelial composite application can be found in a recent review [290].

A few companies are investing in mycelia as the alternative to traditional and plastic materials. Mycelia materials can be 3D printed. There are also examples of injection molding of mycelia materials, where a slurry or liquid aggregate of material containing spores can be injected into a mold of an automotive component shape, with the spores allowed to grow for a specific amount of time. This presents an alternative to plastics used for structural and aesthetic purposes, and use processing methods that are already widely accepted [307–309]. There is also promise in developing insulative packaging materials [310]. Ecovative Design LLC launched MycoComposite™ which it licenses to small companies for use as packaging materials. A patent review covering the period 2009–2018 reveals that 47 patents were awarded for the production and processing of mycelia for various applications, with the majority of the patents in the US and China [289]. During that timeframe, Ecovative accounted for 45% of all patents [289]. Working with Ecovative, students at Massachusetts Institute of Technology tested the production of a living material made from mycelial blocks produced by *Ganoderma sp.* infused with engineered *Bacillus* species [311]. The resulting bricks were melanized by the engineered *Bacillus *[311], providing a proof-of-concept for the co-culture of fungi and bacteria in mycelial material production and opening the path for imparting more complex functions into structural materials [312]. There is also interest in using mycelium-derived foams for automotive applications such as roofs, seats, and dashboards, taking advantage of the low weight and reasonable flame resistance of these materials [289, 313].

A key advantage of materials that contain living organisms is the ability for the growth of those organisms to heal small tears and thereby seal ruptured packaging. Recent work by Shen and co-workers 3D-printed a mixture of chitosan, cellulose, and coffee grounds. After inoculating this mixture with fungus, it was found that while the tensile and compressive strength of the material was reduced by the growth of mycelium, this growth resulted in a material that was able to heal gaps of up to 8 mm. Growth of the mycelium also resulted in a more-hydrophobic surface that absorbed less water. The resulting mycelium-based material had an elastic modulus exceeding 150 MPa, a 15-fold improvement over other materials made of fungal mycelia. The parameters of the printed material, such as sample geometry, toolpath, and the fiber orientation affected by both these parameters, also had a noticeable effect on the yield strengths of the resulting material [314]. Elsacker and co-workers also recently published work on self-healing fungal materials [315], and Sinha and co-workers have also published work on living fiber dispersions, which are capable not only of self-healing, but also of altering their properties by changing humidity, enabling easier end-of-life management of the material [316]. These sorts of fungal engineered living materials, where mycelia continue to actively grow in a matrix, are promising from the standpoints of sensing, self-healing, and continuously tunable properties.

#### Fungal electronics

Fungal-based materials have recently been explored for their suitability in power and electronics applications. Fungi are attractive for potentially making biodegradable sensors that could be scattered in a field to gather data over a period of time and then allowed to degrade in situ. This field is rapidly developing and has been reviewed elsewhere [317], but here we present a few particularly exciting recent findings. None of the examples presented here are yet commercially produced, but they are interesting demonstrations of what could be done with fungal materials in the world of electronics.

Danninger and co-workers grew *Ganoderma lucidum* on a polyethylene sheet which separated the fungus from a hardwood substrate, leading to a “skin” of mycelium that could then be harvested. The resulting “skin” was metalized using physical vapor deposition, and this metalized skin could then be integrated into a circuit board, resulting in a flexible electronic sensor. It was found that the length of time the skin was allowed to grow affected the homogeneity and other physical properties of the resulting device, with growth going on for too long resulting in a mycelium skin for which no continuous metal film could be formed. Through laser ablation, it was possible to precisely control the shape of the metal coat. The authors suggest using the material for flexible batteries, and as a sensor [318]. Fungal skins can also be used as a conductive surface to deliver electrical signals to robots [319], and can also be used to make wearable sensors [320]. A biohybrid model has been developed to convert rhythmic voltage spikes generated by fungal mycelium into signals that control the movement of a soft, starfish-inspired robot and a wheeled robot. The robot was also capable of responding to electrical signals from the fungi which were triggered by changes in light intensity. This demonstrates the potential of using the electrophysiological activity of fungi in robotic applications [321].

Li et al. have demonstrated that advanced electronics with high functionality can be fabricated by integrating conventional nondegradable circuit components with fully degradable, mycelium‐based composites. Their work encompasses the development of printed circuitry as well as the fabrication of both a battery and a sensor. Thermogravimetric analysis (TGA) conducted on various fungal skin compositions indicated stability beyond 250 °C, which permits the soldering of circuitry directly onto the substrate. In addition, the study presents the application of the mycelium composite as a battery separator and packaging material. Importantly, the performance characteristics of the composite were found to vary with the battery chemistry employed, as evidenced in demonstrations using zinc–carbon cell chemistry. The conductivity of the mycelium‐based separator was comparable to that of Li‐ion cell batteries, with MacMullin numbers reported as low as 6.7. Furthermore, the authors successfully integrated proximity and humidity sensors by combining a printed, mycelium-based sensor board with a mycelium-derived battery that powered a communication module featuring an embedded impedance sensor. This work represents an impressive and comprehensive set of demonstrations for mycelium-based electronics that do not include living components [322].

Having a degradable sensor, but a conventional battery that has to be recovered, would seem to be counterproductive. There is therefore interest in creating degradable microbial fuel cells using fungus. A novel fungal-based, biodegradable microbial fuel cell was recently reported by Reyes and co-workers: *S. cerevisiae* was incorporated into a cellulose hydrogel doped with carbon black, graphite, and other nutrients to print an anode, and the WRF *T. pubescens* was incorporated into the same sort of hydrogel to print a cathode. The *S. cerevisiae* produces dehydrogenases as oxidizing agents, and the *T. pubescens* is shown to produce a laccase as a reducing agent, enabling the function of the device. The 3D printability of the device is particularly noted, showing that the device could be made with a number of different form factors [323]. Four of these fuel cells in parallel provide sufficient energy to power a sensor for 65 h. Combining the approaches of Danninger and Reyes provides a very interesting possibility: a degradable in situ sensor coupled with a degradable power source would be environmentally friendly and potentially more economical compared to having to recover both the sensor and the power source for safe disposal. However, both sorts of devices still contain metal, and the amount of metal left behind after natural degradation of the organic components should still be factored in when trying to make such an eco-friendly sensor package. Some recent research has focused on the propagation of electrical signals in living materials which have not been modified by inorganic fillers [324–326], and further understanding of the properties of these unmodified substrates will be critical for the construction of fully-biodegradable fungal mycelium-based electronics and computers.

Development of fungi-based applications for electronics development is underway as demonstrated by the several examples discussed above in this section. Nevertheless, these applications are at a proof-of-concept stage and would require intense research and development to enable generation of ready-to-deploy prototypes. The developed models, however, provide solid impetus to applying mycelia-based systems to real-world technologies. Considering the interest in using fungi in these applications and its feasibility, it is expected that this technology will gain traction from joint academic-industry ventures to help attain reasonable level of commercial maturity in the next 5–10 years.

Mycelium-based materials hold significant promise due to their renewability and their potential to be produced using waste materials. However, their pathway to market success may lie through capturing the same advantages that polymers have, such the ability to make the material economically, the ability to shape the material into various desired forms, and the ability to tune the properties of the material to meet the requirements of diverse applications. While downstream processing of fungal materials will allow some of these important milestones to be achieved, genetic modification of the organisms is likely to be required to achieve all of them.

### Food products

Fruiting bodies and mycelium of fungi can both be used for protein-rich food products. Fruiting bodies (stem and cap) are the collection of hyphae that make up the multicellular structure housing spores. The common button mushroom *Agaricus bisporus* is a familiar example of a fruiting body, but not all filamentous fungi make such structures. In this section, we focus on the production and use of the mycelium.

Fungi are becoming more prevalent in the meat substitute market due to their low fat, high protein and high fiber contents [327]. The growing availability of consumer meat substitutes is driven by such factors as consumer environmental consciousness, ethical impacts of meat production, population growth, and the accessibility of sustainable solutions such as insect and microbial biomass protein. One study found that consumers generally consider environmental benefits of cultured meat products to be a positive, though it is not the sole driver [328]. Individual consumers balance positive drivers, such as environmental and health benefits against negative drivers such as differences in taste, smell, texture, and nutritional content. Perceptions around safety and specific nutritional content such as vitamins, minerals, fiber, cholesterol, and essential fats also impact acceptance. These factors combined contribute to food and food technology neophobia, the tendency to avoid new foods and food technology [329]. Another key factor is overcoming the negative stigma of “mold” in regard to food and other biomaterials [330]. Cost has a major impact as these new products tend to require premium pricing. However, consumption of mushrooms is commonly accepted, meaning that with the right marketing, there is likely to be wider acceptance of fungal-based foodstuffs.As these products have come to market, there will have to be discussions about how governments should write dietary guidelines and recommendations for a population that consumes more mycoprotein, with such a discussion recently being the subject of a 2024 roundtable at the British Nutrition Foundation which was summarized in a recent article [327].

Another potential concern is that novel proteins (such as fungal proteins) in the diet might cause allergic reactions. While pollen food allergen syndrome has been well-characterized, the similar fungal food allergen syndrome has had limited study [331]. Though rare, prior sensitization to airborne fungal allergens can result in an immune response triggered through cross-reactivity with ingested mycoprotein, leading to an array of symptoms from mild gastrointestinal stress to anaphylactic shock [331]. The rare occurrence of these incidents happens primarily in subjects with known fungal allergies.

Bacteria, yeast, and fungi have been used for biomass protein products used to feed fish [277, 332], livestock [285], and humans [276, 333]. Filamentous fungi are particularly excellent for these applications because they grow quickly, generate large quantities of biomass, and due to their morphology are able to be easily extracted from the feedstock [276]. Fungi have also long been used in traditional foods, or consumed directly (tempeh, mushrooms), which might ease efforts to make fungal-based foods more widely accepted.

Beginning in the 1980s, consumer food companies began introducing new fungal biomass protein products to the global market. The amino acid profiles of fungal biomass protein align with the Food and Agriculture Organization/World Health Organization standard for human nutrition [333, 334]. Table 3 summarizes some of the mycelia-based protein products currently on the market. The Good Food Institute, a non-profit organization, released a State of the Industry report in late 2020 [335]. This report summarizes the innovations in fermentation that are giving rise to a new era in the food sector, specifically as it pertains to alternative protein sources. Quorn, founded in 1985, was the leading myco-based food producer world-wide, and had a nearly exclusive market up until around 2020, when newer startups began emerging [329]. The total list of companies that were founded between 2017 and 2020 and use mycelium for alternative protein options include: Ecovative-Atlast™, Mycorena, Meati Foods, MycoTechnology, Nature’s Fynd, ENOUGH, Enifer, Foods Myco Laboratory, Fybraworks Foods, Kernal Mycofood (now Eternal Bioworks Corp), Kinoko-Tech, Mushlabs, Prime Roots, Pura, and The Protein Brewery. Overall, the alternative protein industry is extremely young and is currently experiencing tremendous growth raising a record $3.1 billion in investments in 2020, with fermentation companies accounting for $590 million [335]. Fungal protein processing and composition has recently been reviewed elsewhere [336]. In their 2023 sustainability report, ENOUGH reports producing 1.3 tons of mycoprotein per hour, the equivalent of five cows worth of meat, from continuous fermentation in a 30-m-tall fermenter [337]. This amounts to 10,000 tons per year at an annualized rate. The mycoprotein production industry will need to grow rapidly to provide viable protein alternatives to meet the global meat demand of ~600 million tons per year (Food and Agriculture Organization of the United Nations (2024); Population based on various sources (2024)—with major processing by Our World in Data. https://ourworldindata.org/). Efforts are also underway to make mycoprotein have a taste and texture closer to that of actual meat. Meat substitutes such as the IMPOSSIBLE burger have relied on the introduction of heme protein, to provide the red color and flavor associated with meat. *A. oryzae*, a fungus which is already used in fermented foods, was recently modified to produce heme to more closely approximate the flavor and color of meat, as well as the antioxidant ergothioneine, further enhancing the nutritional value of the meat substitute [338]. Mycoprotein provides a potential avenue for point-of-need protein production such as would be needed for deep space missions, which highlights the versatile aspects of mycoprotein for human consumption.

**Table 3:** Food sector companies leveraging fungal fermentation

Company name	Year	Product name	Type of fermentation
Marlow Foods Ltd	1985	Quorn	Solid state
MycoTechnology	2012	FermentIQ™	Submerged
ENOUGH (3FBIO Ltd.)	2015	ABUNDA ®	Submerged
Ecovative Design LLC, Atlast™ Food Co	2019	My	Solid state
Mycorena	2020	Promyc™	Submerged
Enifer	2020	PEKILO ®	Submerged
Meati Foods/Emergy Foods	2021	Mycelium steak	Submerged
Nature’s Fynd (Sustainable Bioproducts LLC)	2021	Fy™	Interface

In 2002, the US Food and Drug Administration (FDA) determined mycoprotein to be GRAS (https://wayback.archive-it.org/7993/20171031023444/https://www.fda.gov/Food/IngredientsPackagingLabeling/GRAS/NoticeInventory/ucm154623.htm). However, in some instances consumers report adverse effects [339]. Quorn currently uses the following package warning “Mycoprotein is a mold (member of the fungi family). There have been rare cases of allergic reactions to products that contain mycoprotein.”

It is currently unknown and understudied what the root cause of the adverse reactions are in some consumers. While novel food sources present new opportunities to combat food scarcity and hunger across the globe, they also risk introducing potential new allergens into the global food supply. While currently fungi-based foods are not genetically engineered, there may be interest in developing genetically-modified foods for consumption, like current food crops. However, this presents a potential allergenic hazard as genetic modification of organisms can result in the introduction of non-native proteins in food sources, some of which may be allergenic, therefore a platform for hazard and risk assessment for genetically engineered organisms is needed to identify potential new allergens in novel food sources (US Federal Register Docket No. 92N-0139, Vol 57, No. 104, May 29, 1992). In 2001, the United Nations and World Health Organization recommended looking for 6 amino acid peptide matches to known existing allergen in addition to immunoglobulin E (IgE) binding studies [340]. However, it is not practical, even with current access to genomics and proteomics data, to assess every predicted protein of organisms intended for food use in IgE binding studies. In addition, relying on IgE studies was later shown to be non-predictive [341], and the current approach looks for >35% identity over a minimum of 80 amino acids in addition to considering the E-score [342]. A recent study by Abdelmoteleb et.al performed a whole genome analysis to assess the current process for allergen identification [343]. The authors compared housekeeping proteins across 23 species and found moderate to low alignment with minor allergens identified in TheAllergenOnline.org (AOL) database. Since housekeeping genes are conserved across eukaryotes, they will be found using the current criteria of >35% identity across at least 80 amino acids and E-scores. In addition, major allergens are not conserved in sequence or structure and therefore are not identified using the current criteria. The authors conclude by suggesting the current criteria over-predicts allergens and suggest increasing the identity score or lowering the E-score criteria [343]. While protein abundancy and potency also need to be assessed for new organisms intended for food use, the whole genome assessment is a valuable bioinformatic approach for the microbial biomass protein community. It remains likely that patients who are allergic to fungi may react adversely to mycoprotein due to shared allergic determinants [331]. Individual safety determinations for novel protein sources should be mandatory even if the broad category of mycoprotein is GRAS [344]. With the anticipated increase in protein transition to more sustainable or environmentally friendly protein sources, there is currently a major need for technology to assess allergenicity of fungal derived protein and food products.

In 2016, an engineered white button mushroom (*A. bisporus*) was the first genetically modified organism (GMO) food product which did not require United States Department of Agriculture (USDA) approval [345]. The United States Environmental Protection Agency only regulates GMOs associated with pesticide treatment and the FDA considers GMOs and GMO ingredients to be safe as long as they follow the standard food safety guidelines (www.fda.gov). Whereas the USDA prohibits the introduction of foreign DNA into a host via CRISPR, the modification of native genes using CRISPR, in a transient manner, is an apparent exception to the regulations. Therefore, the new white button mushroom engineered by Yinong Yang of Pennsylvania State University to prevent browning, is not subject to USDA regulation and may become a commercially sold product [346, 347]. While several genetically modified organisms have been able to sidestep USDA regulations in the past decades, this is the first example of CRISPR modification.

Genetically modified fungi are not yet used in the production of biomass protein, but synthetic biology has been used to metabolically engineer yeast for desired performance in beer and wine production. For example, Berkeley Yeast applied metabolic engineering to get *S. cerevisiae* to produce aromatic monoterpene molecules that give beer its hoppy flavor [348]. The same approaches are likely to be applied to filamentous fungi used in biomass protein production in the future. Metabolic engineering together with genome mining for novel enzymes for substrate breakdown, and/or bioprospecting for new strains, will be leveraged by synthetic biologists to expand the capabilities (and flavors) of filamentous fungi in the food sector.

### Distributed biomanufacturing and future applications

Sustainable and recyclable building materials are also appealing in situations where resources are limited and the logistics of resupply is difficult, such as in the exploration of outer space, or in healthcare settings in the limited-resource environments of the developing world [349]. The ideal scenario for far-reaching, deep space missions would rely on the development of in situ resource utilization methods, closed-loop life support systems, and protection from extreme environments [350]. Microorganisms provide a low weight payload and high biomass generating resource that is already leveraged extensively in biotechnology. Many space flight experiments have tested the production of commodity chemicals such as antibiotics from yeast and bacteria [350]. Filamentous fungi are notorious as ubiquitous members of the microbiota associated with spacecraft environments, and have been found as contaminants in clean rooms and assembly facilities [351]. Unlike bacteria, bacterial spores, and yeast, filamentous fungi are less prone to ultraviolet (UV) induced DNA damage, an important advantage in the high-radiation environment of outer space [352]. Current research on the International Space Station studies the growth and survival of various species of fungi in space, as foundational work that can be leveraged in developing further biotechnology applications [353].

Besides understanding fungi as contaminants, NASA is interested in leveraging mycelial materials as grow-on-site structures for the colonization of off planet environments [354]. Pilot studies for this concept involve developing fundamental understanding of mycelial growth as part of a consortia with algae and bacteria, which together create a symbiotic environment supporting growth of the structure [354]. Tunable material properties and self-growing, self-healing structures are ideal for austere environments where resources are scarce and payload weights are limited. The concept of fungal biotechnology in space has been reviewed elsewhere [350], and the concept is still emerging, but the ability of fungi to grow on a wide variety of materials, starting from some relatively light spores, makes them particularly attractive for applications where weight is at a premium.

## Conclusions

Filamentous fungi have historically been used for a wide range of industrial applications and remain a promising set of organisms for producing a wide-ranging array of commercially relevant products, such as organic acids, lipids, secondary metabolites, and enzymes. Aside from what they produce, the biomass of the fungi themselves is potentially valuable in building renewable materials from waste streams, and in growing new sources of food. The ability of fungi to convert waste products such as wood and agricultural waste to valuable products means that they are likely to play an important role in the development of circular economies in the future. However, the systematic use of synthetic and molecular biology tools to improve fungal properties is still in its infancy. This is further challenged by the constant discoveries of new, uncharacterized fungi with unique properties, in the environment. With increasing development of new tools for genetic manipulation of filamentous fungi, and with increasing concerns about sustainability, more genetic modification of fungi is likely to soon solve persistent challenges facing humanity, such as how we dispose of waste, what we eat, what we wear, and what we use to build our environments.

## Data Availability

No datasets were generated or analysed during the current study.
